# Comparing transfer learning to feature optimization in microstructure classification

**DOI:** 10.1016/j.isci.2022.103774

**Published:** 2022-01-15

**Authors:** Debanshu Banerjee, Taylor D. Sparks

**Affiliations:** 1 Metallurgical and Material Engineering Department, Jadavpur University, Kolkata, West Bengal 700032, India; 2 Department of Materials Science and Engineering, The University of Utah, Salt Lake City, UT 84112, USA

**Keywords:** Computer modeling, Materials science, Computational materials science

## Abstract

Human analysis of research data is slow and inefficient. In recent years, machine learning tools have advanced our capability to perform tasks normally carried out by humans, such as image segmentation and classification. In this work, we seek to further improve binary classification models for high-throughput identification of different microstructural morphologies. We utilize a dataset with limited observations (133 dendritic structures, 444 non-dendritic) and employ data augmentation via rotation and translation to enhance the dataset six-fold. Then, transfer learning is carried out using pre-trained networks VGG16, InceptionV3, and Xception achieving only moderate F1 scores (0.801–0.822). We hypothesize that feature engineering could yield better results than transfer learning alone. To test this, we employ a new nature-inspired feature optimization algorithm, the Binary Red Deer Algorithm (BRDA), to carry out binary classification and observe F1 scores in the range of 0.96.

## Highlights

• A dataset comprising two categories of micrographs has been prepared
• Transfer learning has been implemented for micrograph classification
• To improve upon the classification accuracy, we perform feature engineering
• Feature engineering has been performed using Binary Red Deer Algorithm

## Graphical abstract

**Figure:**
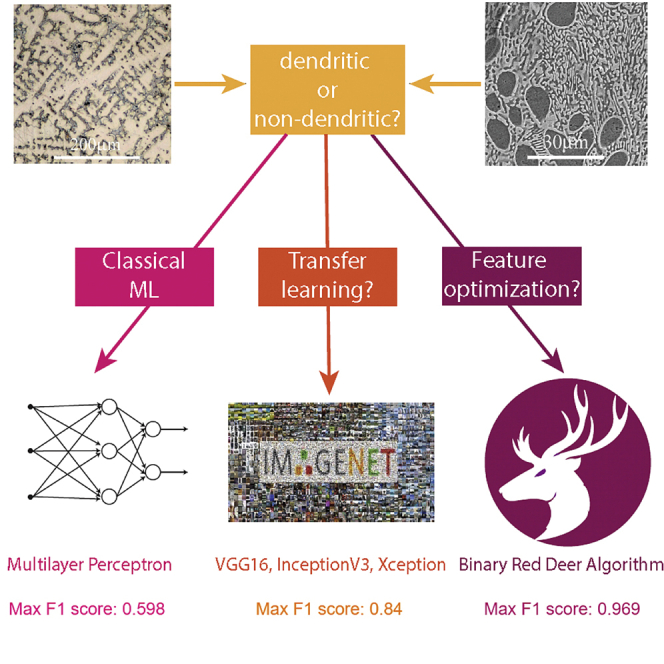

## Introduction

Materials science is centered around the concept of understanding, extracting, and exploiting relationships between structure, property, processing, and characterization of materials. Images of a material's crystal or microstructure can be invaluable in establishing structure-processing-property linkages. However, the human eye itself can only resolve objects as small as ∼ 50 μm. Investigating smaller details than this requires microscopy like optical microscopy, transmission electron microscopy (TEM), scanning electron microscopy (SEM), atomic force microscopy (AFM), and others. Each of these techniques offers different resolution limitations along with other advantages and disadvantages.

Analysis of microstructure images can yield an enormous amount of information about a material! For example, some alloys exhibit a dendritic structure characterized by the presence of myriad snow-flake-like dendrites that form during the solidification process of casting molten metals. Dendrites can form when the interface of the solid cast and the liquid alloy has a lower temperature as compared to the remaining melt resulting in a temperature gradient known as constitutional undercooling (Hurle, 1961). Because dendrites can modify the mechanical properties in beneficial or detrimental ways (Wang et al., 2003), it is important to classify microstructures of materials as dendritic and non-dendritic.

Traditionally, humans have leveraged training and extensive domain knowledge to interpret and categorize microscopy images. This poses challenges for interdisciplinary research as well as automated, human-out-of-the-loop experimentation. Moreover, even highly trained humans are still prone to errors and bias during materials characterization. This gave rise to the implementation of computational methods on the study of dendritic microstructures which started back in 1998 to focus on the evolution of the said microstructures using phase-field modeling (Provataset al., 1998). Later attempts have been made on three-dimensional reconstruction of microstructures captured from different microscopes which have helped in getting better insights about the mechanical properties (Alkemper and Voorhees, 2001; Uchic, 2011). But all these works were simulation-based and time-consuming.

An alternative to physics-based models is statistical, data-driven models that trade accuracy for speed by leveraging correlations and patterns in data. Machine learning models have been used extensively to analyze various microstructural morphologies over the last two decades (Yang et al., 2021). One such example is the development of optimal morphology derivation from a given microstructure using Bayesian optimization and kinetic Monte Carlo simulation (Tran et al., 2020). Transfer learning has been deployed for microstructure reconstruction and structure-property predictions (Li et al., 2018) whilesupport vector regression and multilayer perceptron (MLP) is used to predict information related to dendrite formation to improve high-temperature creep and fatigue resistance (Jiang et al., 2018). Different types of industry-relevant titanium alloy microstructures can now be classified using convolution neural networks (Baskaranet al., 2020). A particle swarm optimization algorithm has been deployed to classify the casting techniques resulting in dendritic and non-dendritic microstructures in aluminum metal matrix composites (Shabaniet al., 2015). Advanced computer vision techniques have even been utilized to generalize microstructure morphology classification beyond individual alloy systems (Impoco and Tuminello, 2015; Chowdhuryet al., 2016; Nikolićet al., 2021).

In this work, we turn to a recently reported, nature-inspired algorithm called Binary Red Deer Algorithm (BRDA) (Fathollahi-Fard et al., 2020) to implement feature engineering to classify microstructural morphologies as dendritic or non-dendritic. For reference, we also present a comparative study between the transfer learning approach and the BRDA feature engineering approach in classifying microstructural morphologies. We show that BRDA outperforms advanced transfer learning techniques and has promise in the evolving field of computer vision in materials informatics.

## Results and discussion

**Figure 1:**
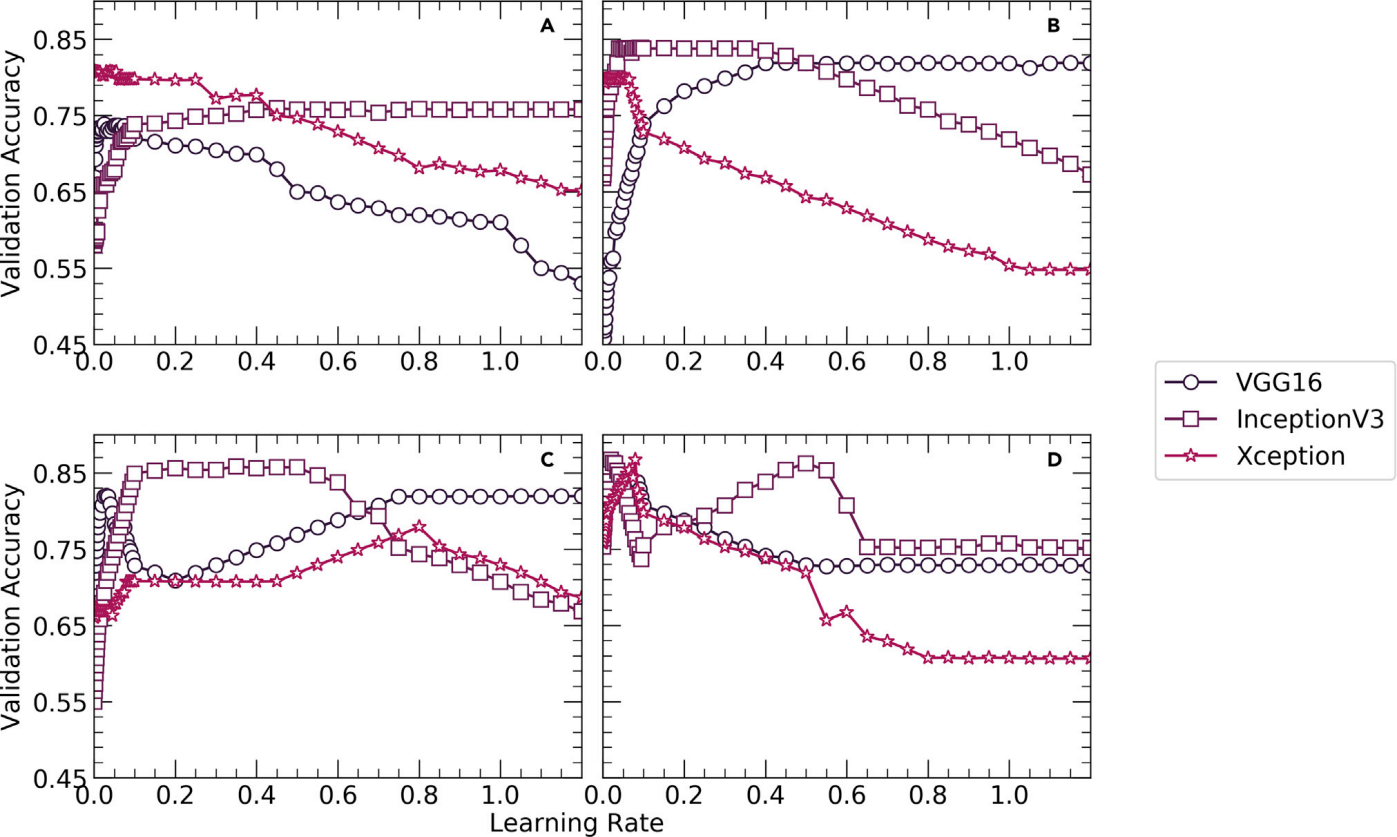
Plots showing the variation of validation accuracy with learning rate using (A) NAG, (B) Adagrad, (C) RMSProp and (D) ADAM optimizers for all three networks

**Figure 2:**
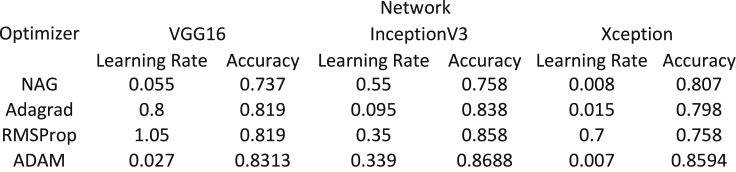
The maximum validation accuracy and the corresponding learning rate obtained from using NAG, Adagrad, RMSProp and ADAM optimizers for all three networks

The code to build the transfer learning and feature engineering-based models has been developed predominantly by using two Python libraries, *TensorFlow Core v2.5.0* and *Scikit-Learn 0.24.2*. The optimal learning rate for each of the four optimizers: NAG, Adagrad, RMSProp, and ADAM is determined experimentally on a trial-and-error basis. Figure 1 shows the variation of validation accuracy with the learning rate for all four optimizers across all three network architectures. Using NAG optimizer, the validation accuracies for VGG16 and Xception decrease continuously starting from 0.74 to 0.81, respectively, whereas the same for InceptionV3 increases continuously up to 0.77. This shows that the NAG optimizer is most efficient in the case of Xception. Using Adagrad optimizer, there is a continuous decrease in the validation accuracies for Xception and vice versa for VGG16 and InceptionV3. But InceptionV3 shows predominantly higher validation accuracies as compared to VGG16 and Xception particularly when the learning rate is between 0.02 and 0.42. Using RMSProp optimizer, similar trends are observed in cases of InceptionV3 and Xception where the validation accuracies increase up to a maximum value followed by a sharp decrease while a reverse trend is observed in the case of VGG16. However, InceptionV3 has predominantly had higher validation accuracies as compared to VGG16 and Xception. Using ADAM optimizer, InceptionV3 and Xception show irregular trends whileVGG16 has a parabolic decrement in validation accuracies to the learning rate. However, the maximum validation accuracies obtained with the ADAM optimizer for all three networks surpass the maximum accuracies obtained with the remaining three optimizers as shown in Figure 2.

**Figure 3:**
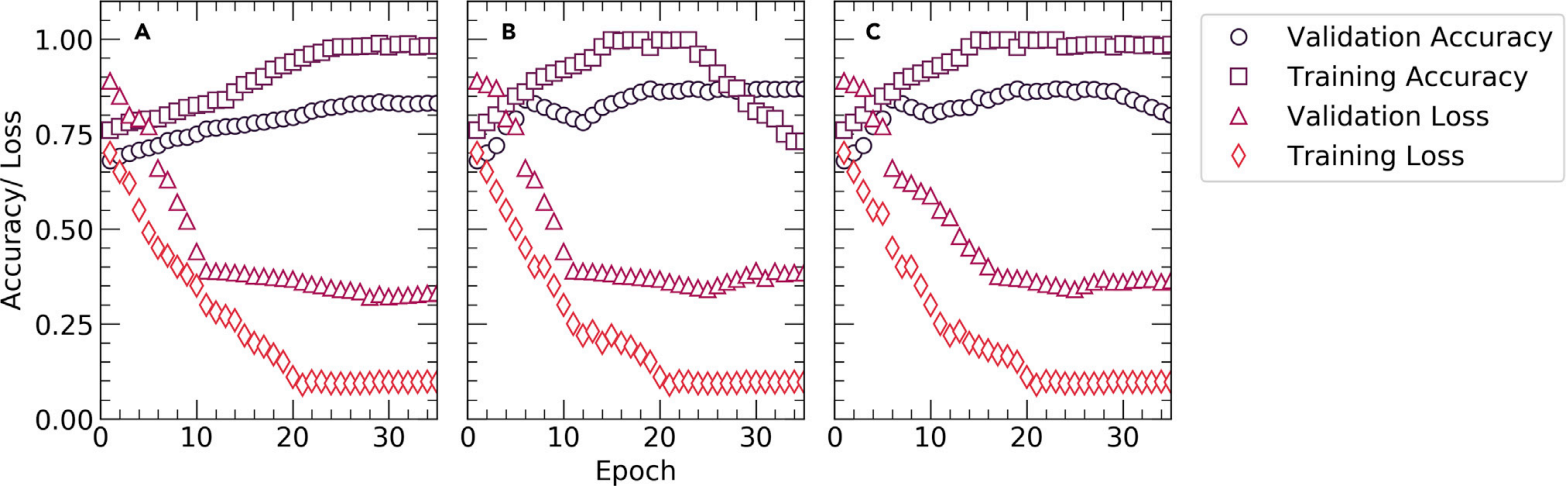
Schematic diagram showing The performance of (A) VGG16, (B) InceptionV3 and (C) Xception networks on the augmented dataset

**Figure 4:**
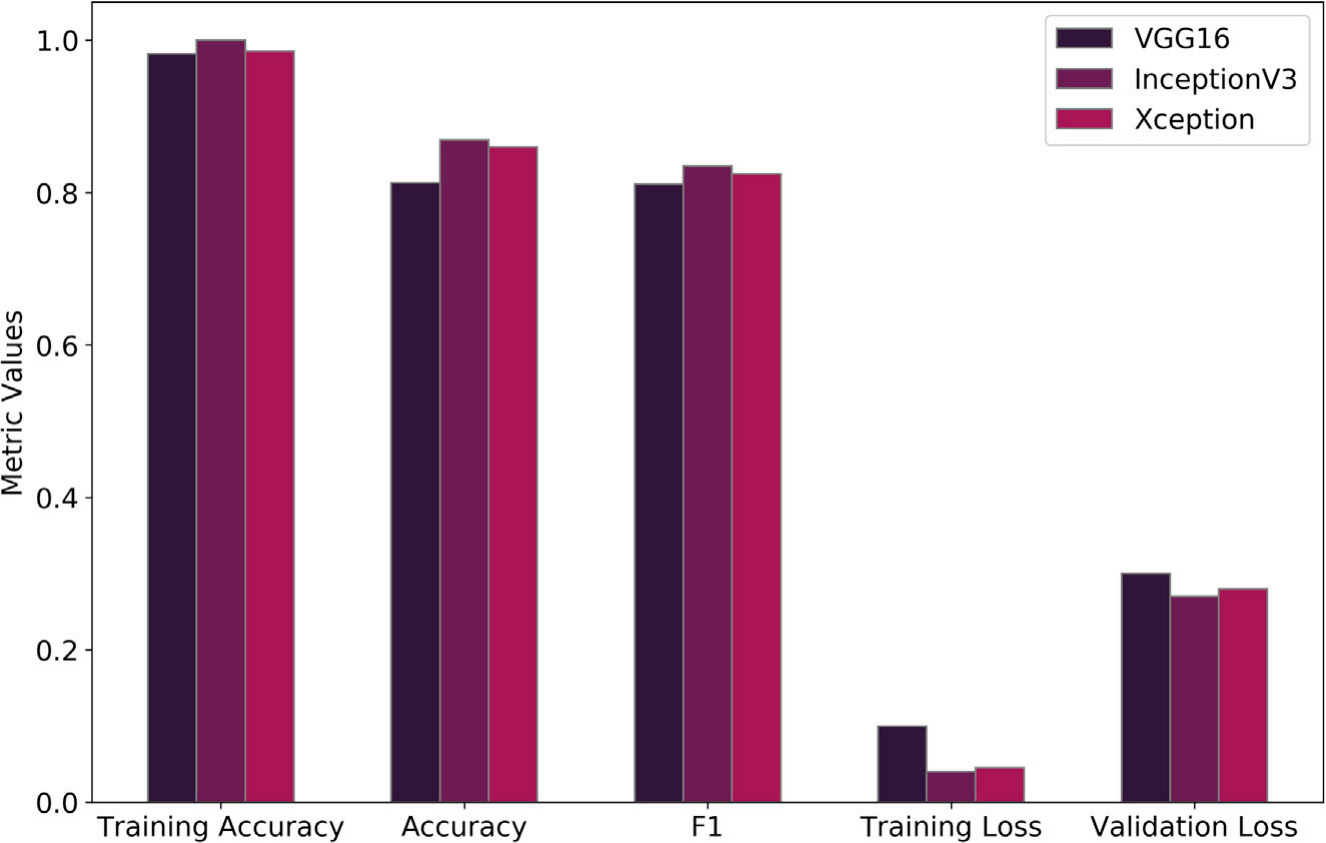
Schematic diagram showing the comparison between the accuracies and CCE losses for training and validation of the final pre-trained network architectures

Therefore, transfer learning with the proposed network architectures using the ADAM optimizer provided the best micrograph classification results with validation accuracies equal to 0.8313, 0.8688, and 0.8594 for VGG16, InceptionV3, and Xception, respectively, where the learning rates for the ADAM optimizer are 0.027, 0.339, and 0.007 for VGG16, InceptionV3, and Xception, respectively. Early stopping has been deployed in the case of training the pre-trained networks to minimize the number of epochs and obtain the optimum results (Prechelt, 2012). Consequently, *VGG16*, *Inception3*, and *Xception* were stopped after 35, 37, and 43 epochs, respectively using the ADAM optimizer where the training failed to reach any maxima beyond this point. Figure 3 delineates the epoch-wise training accuracies, validation accuracies, CCE training loss, and CCE validation loss for each of the three networks. Early stopping gives us validation accuracies of 0.8313, 0.8688, and 0.8594 as shown in Figure 2. and validation losses of 0.3, 0.27, and 0.28 for VGG16, Inception3, and Xception, respectively, and the corresponding training accuracies for these networks are 0.9819, 0.9997, and 0.9856 while the training losses are 0.1, 0.04, and 0.045, respectively as shown in Figure 4. These results indicate that even the best combination and frozen and unfrozen layers fail to provide a generalized binary classifier due to overfitting.

**Figure 5:**
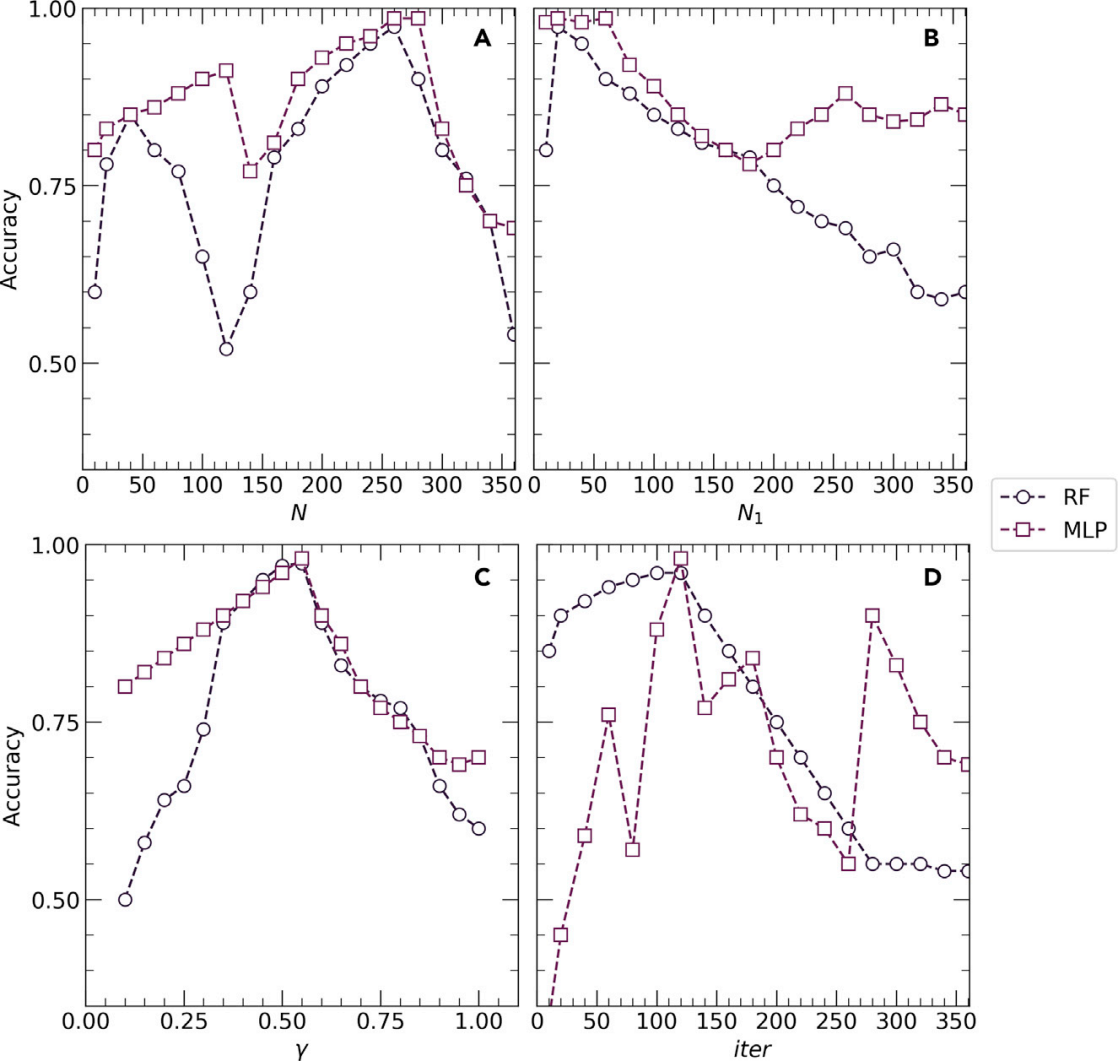
Schematic diagram showing the experimental determination of the parameters of BRDA (A) RD population (N) (B) Number of male RDs (N_1_) (C) Ratio of the number of commanders to total number of male RDs () and (D) Total number of iterations for which BRDA is executed (iter)

**Figure 6:**
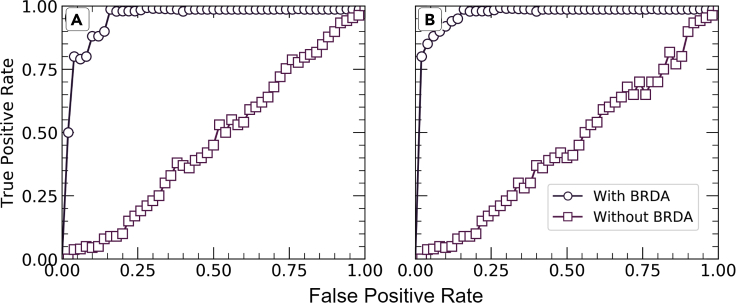
Performance of (A) RF and (B) MLP classifiers with and without using BRDA

BRDA used for feature optimization has several parameters as shown in Table 1 which need to be specified at the start of the algorithm. The optimum values of these parameters have been determined experimentally following a trial-and-error approach. Each of these parameters has been plotted to the corresponding accuracies obtained by the RF and MLP classifiers as shown in Figure 5. From the independent plots, we can determine the optimum values of these parameters. While determining a particular parameter, the remaining parameters are set to arbitrary values. This implies that each of these parameters if set to their optimum values, can alone provide a nearly optimized subset which can lead to accurate classification results which are close to the actual classification results when all the parameters with their optimum values are combined. Therefore, a single parameter itself can bring a significant change to the entire model performance, and parameter tuning becomes an important step of experimentation. No measures have been taken during experimentation to eradicate the class imbalance. Data augmentation has been performed to increase number of images per class in order to have a higher number of images for training the RF and MLP classifiers. Consequently, it can be inferred that the class imbalance that exists in the dataset has no effect in the classification results.

**Table 1:** Optimal parameter settings of the proposed FS method called BRDA

Parameter	Meaning	Value
N	RD population	260
N1	Number of male RDs	20
ϒ	Ratio of the number of commanders to the number of male RDs	0.57
Iter	Total number of iterations for which BRDA is executed	120

**Figure 7:**
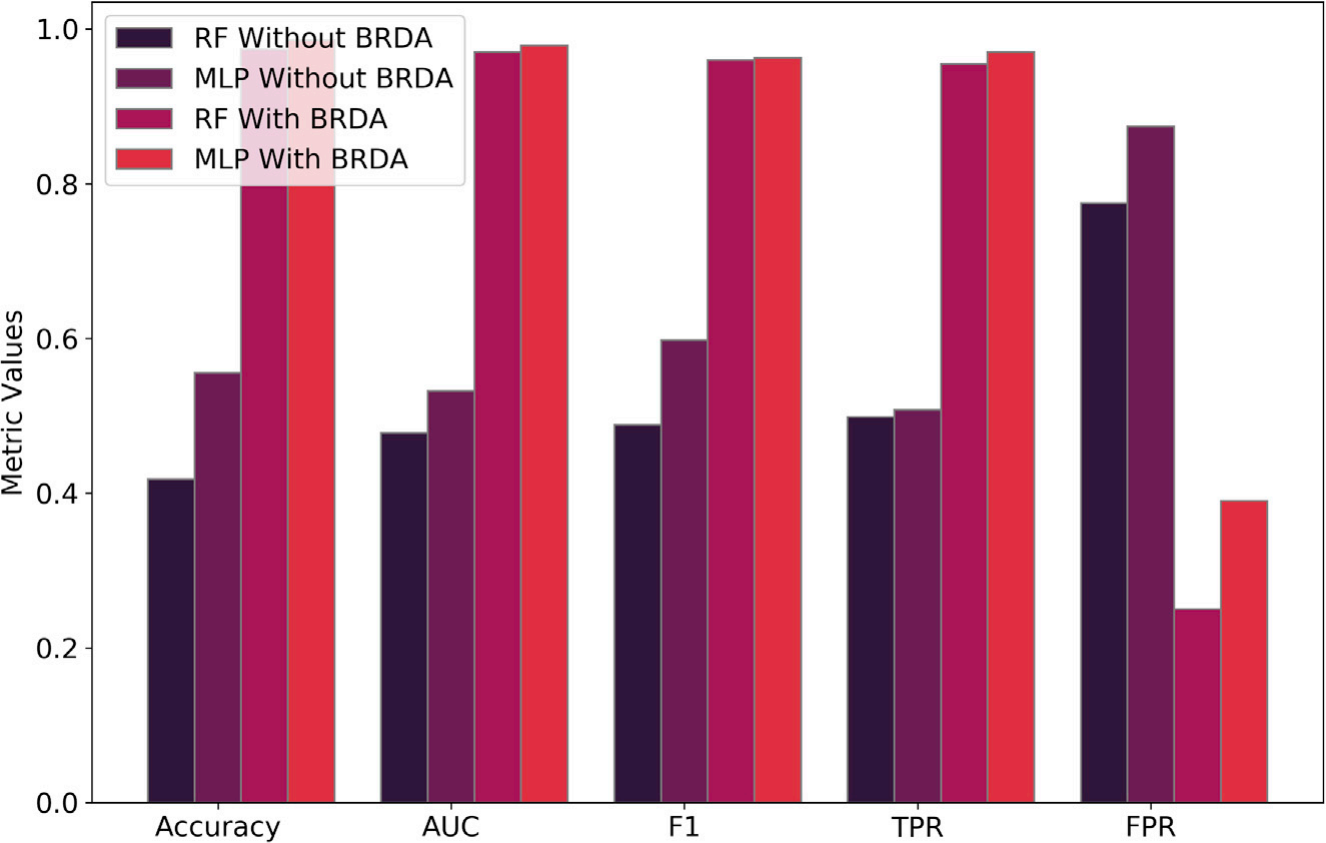
Comparison between the accuracies of RF and MLP classifiers with and without using BRDA

Without BRDA, RF and MLP give poor classification results with accuracies of 0.4198 and 0.5556, respectively. As shown in Figure 6, the area under the ROC curves (AUC) for each of these classifiers without using BRDA are 0.478 and 0.532, respectively. These poor values of classification metrics are an indication of improper classification of dendritic and non-dendritic micrographs which can be seen from the true positive rate (TPR) and false positive rate (FPR) values as shown in Figure 7. TPR values are 0.4987 and 0.5073 and those of FPR are 0.775 and 0.8743 for RF and MLP classifiers, respectively, without using BRDA. However, after deploying the BRDA feature optimization algorithm, we note a drastic improvement in the classification results with accuracies of 0.9735 and 0.9855 and AUC of 0.97 and 0.979 for RF and MLP classifiers, respectively. Besides, the F1 scores for RF and MLPclassifiers are 0.963 and 0.969, respectively, after the implementation of BRDA in contrast to the same without BRDA which are 0.488 and 0.598, respectively. On the contrary, the F1 scores for VGG16, InceptionV3, and Xception network architectures are 0.811, 0.835, and 0.8246, respectively, using the ADAM optimizer. The F1 scores indicate that applying BRDA reduces the number of images falsely classified as the other category significantly. Figure 7 gives a comparative plot depicting the classifier evaluation metrics before and post implementation of BRDA. It has been established from the FPR values that the number of microstructural images which were incorrectly classified without using BRDA has reduced significantly. FPR post implementation of BRDA is 0.39 and 0.25 for RF and MLP, respectively. Figure 6 provides a better understanding of the performance of the classifiers with and without BRDA with the help of ROC curves.

**Figure 8:**
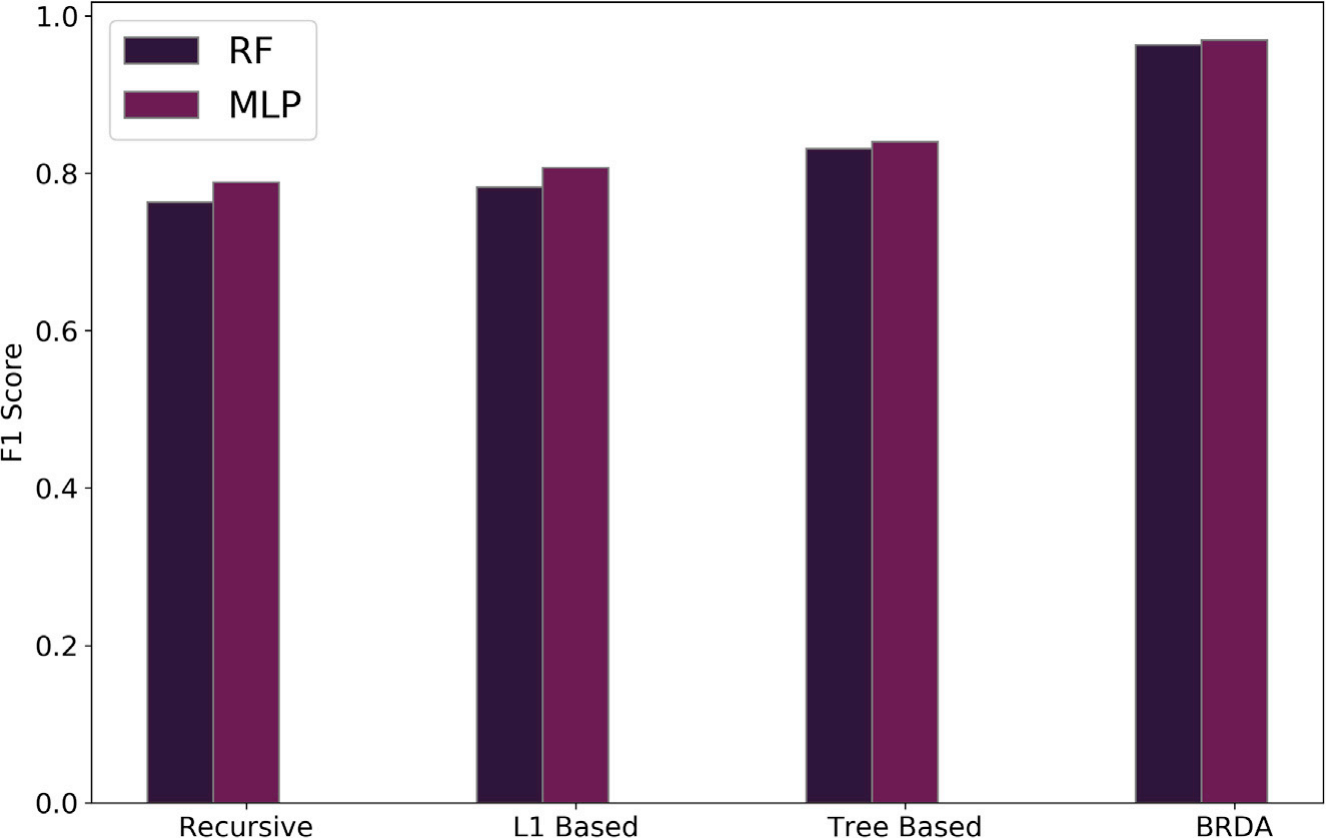
Schematic diagram showing Comparison between the F1 scores of RF and MLP classifiers using BRDA and other feature selection algorithms

**Figure 9:**
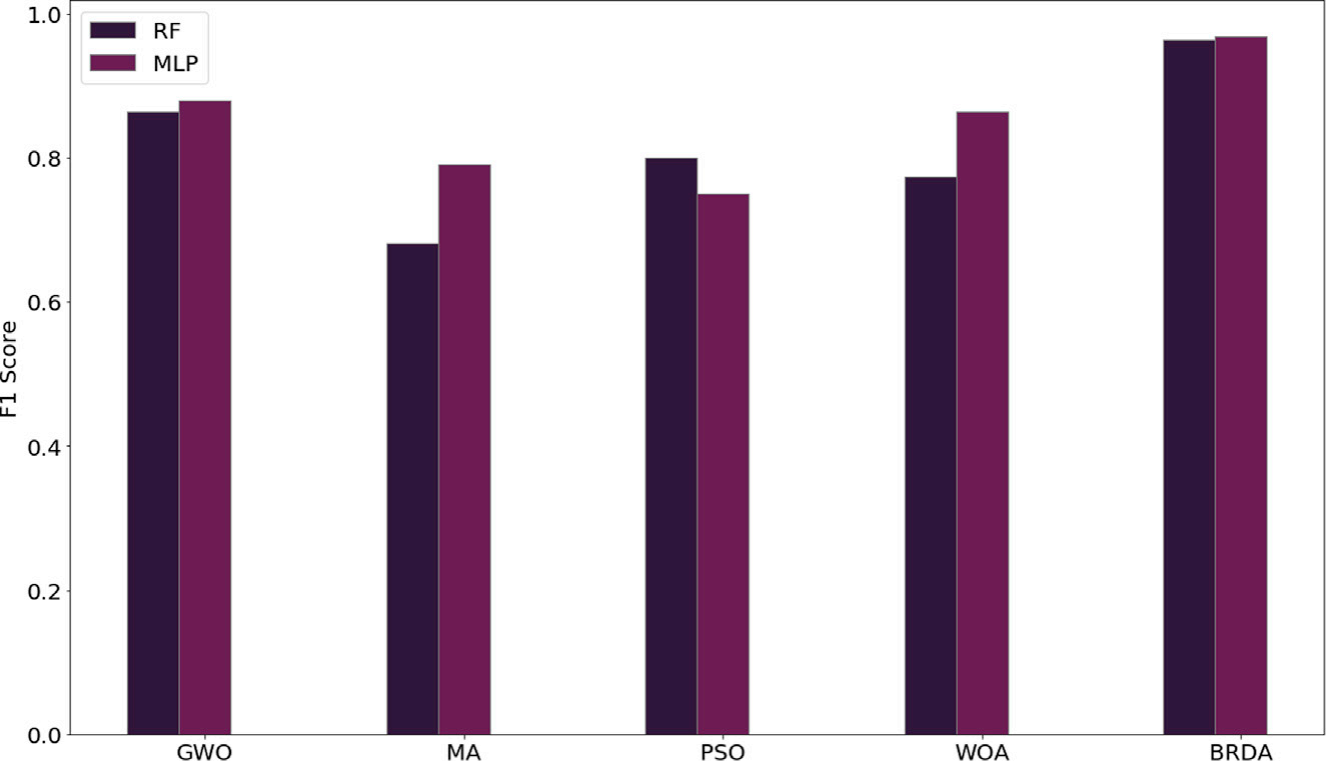
Comparison between the F1 scores of RF and MLP classifiers using BRDA and other nature-inspired optimization algorithms

Therefore, feature optimization using BRDA and using classical machine learning classifiers has proven to be more efficient in characterizing micrographs as dendritic or non-dendritic as compared to transfer learning. The classification results using BRDA have been compared using standard feature selection algorithms available in *Scikit-Learn 0.24.2* library. We have compared our results with recursive, L1-based, and tree-based feature selection methods for both RF and MLP classifiers. Figure 8 shows the variation of F1 scores for RF and MLP classifiers when BRDA and other feature selection algorithms are deployed. F1 scores obtained by using recursive, L1-based, and tree-based feature selection algorithms for RF classifiers are 0.763, 0.781, and 0.831, respectively while the same on MLP classifiers are 0.788, 0.807, and 0.84, respectively. Besides, the obtained results have also been compared with four nature-inspired optimization algorithms: Grey Wolf Optimizer (GWO), Mayfly Algorithm (MA), Particle Swarm Optimization (PSO), and Whale Optimization Algorithm (WOA). A comparison of the F1 scores for each of these feature optimizations and BRDA for both RF and MLP classifiers has been shown in Figure 9. In contrast to the F1 scores obtained for RF and MLP upon using BRDA which are 0.963 and 0.969, respectively, other feature selection algorithms have less efficiency in classifying micrographs as dendritic or non-dendritic. A single micrograph is available in a number of different magnifications in the dataset. So, for a particular micrograph, we have multiple images each with distinct magnifications. It can be inferred that different magnifications of a single micrograph have resulted in high F1 scores when RF and MLP classifiers are implemented following the application of BRDA.

**Figure 10:**
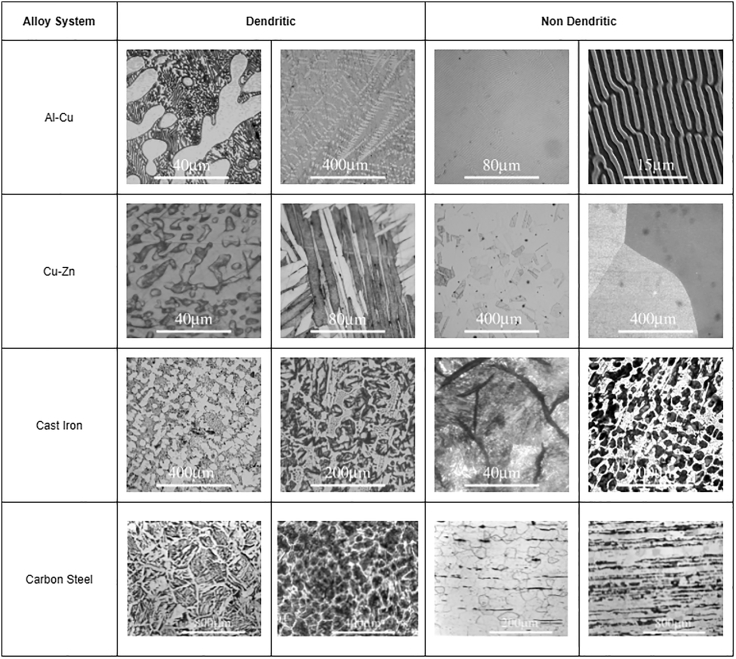
Example micrographs considered in this work

**Figure 11:**
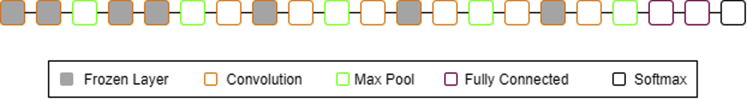
Architecture of *VGG16* network depicting the frozen and unfrozen layers

**Figure 12:**
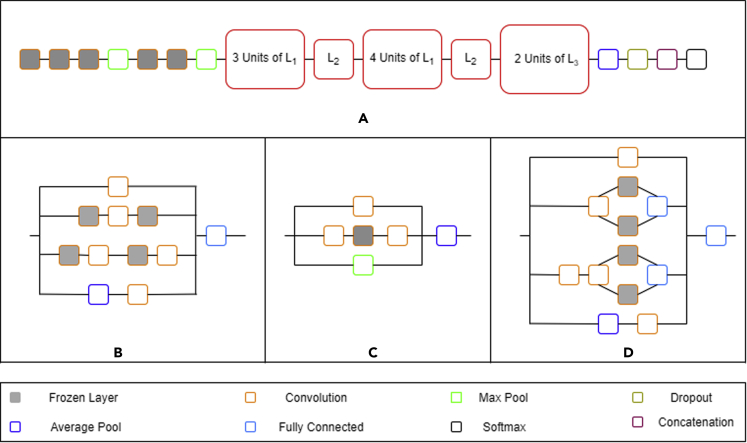
Schematic diagram showing (a) Architecture of InceptionV3 network depicting the frozen and unfrozen layers, (b) Inception module 1 (L1), (c) Inception module 2 (L2) and (d) Inception module 3 (L3)

### Limitations of the study

This work focuses on a comparative study between transfer learning and feature engineering approaches in the classification of dendritic and non-dendritic microstructural morphologies. Transfer learning using *VGG16*, *InceptionV3*, and *Xception* fails to provide proper generalization in microstructural characterization. Consequently, we have utilized a feature engineering approach using a meta-heuristic optimization algorithm called Binary Red Deer Algorithm which improves the performances of two machine learning classifiers drastically. Parameter tuning plays an important role in the optimization of the feature set and therefore impacts the classification results in turn. This work provides an uncommon example of where feature engineering has outperformed transfer learning. Besides, BRDA also has better feature selection ability as compared to some standard feature selection algorithms available in *Scikit-Learn 0.24.2*. However, this work is restricted to 21 different alloys systems. Extending the implementation of BRDA beyond these 21 alloys systems and even to other materials will be our future aim. BRDA, therefore, brings significant promise in the field of materials informatics and we will aim to extend its use beyond materials characterization in future contributions.

## STAR★Methods

### Key resources table

**Table:** 

REAGENT or RESOURCE	SOURCE	IDENTIFIER
**Deposited data**		

Dendritic and non-dendritic micrographs	Dissemination of Information Technology for Promotion of Materials Science (DoITPoMS)	Banerjee et. al., 2021

**Software and algorithms**		

Python	This paper	https://github.com/stochasticmaterialism/Dendritic-Non-Dendritic-Classification

### Resource availability

#### Lead contact

Further information and requests for resources should be directed to and will be fulfilled by the lead contact, Dr. Taylor D. Sparks (sparks@eng.utah.edu).

### Method details

#### Data preprocessing

At first the micrographs are converted to single channel, i.e., grayscale format from three-channel RGB format using *BGRtoGRAY* function available in *OpenCV 4.5.3* (Bradskiet al., 2009) library. Each micrograph has a scale bar at the bottom indicating the magnification at which it has been recorded. The scale bars are not a part of the morphologies of the micrographs and have no significant information towards the classification objective. Consequently, the scale bar potion has been removed by cropping the grayscale micrograph followed by down-sampling to a dimension of 300 × 300. This ensures a uniform 300 × 300 parameter matrix for all the images present in the dataset without encountering any information loss. The down-sampled micrographs are augmented using *ImageDataGenerator* function available in *Keras 2.4.0* (Géron., 2017) to increase the number of images in order to achieve better classification results. Each micrograph is rotated at five different angles, 50°, 100°, 150°, 200°, 250°, and 300° to the original micrograph. This results in six new representations of the initial micrograph. Down-sampling, and data augmentation has been performed separately for the training and test sets to ensure that the six new versions of any micrograph belong to the set, i.e., training or test where the original micrograph is present. Data augmentation gives us a six-fold increase in the number of images with 931 micrographs in the dendritic category and 3108 micrographs in the non-dendritic category. This augmented dataset is now used to build binary image classification models to distinguish the dendritic morphologies from the non-dendritic ones.

#### Transfer learning approach

Three different pre-trained CNN architectures; *VGG-16* (Simonyan and Zisserman, 2015), *InceptionV3* (Zenget al., 2016) and *Xception* (Krishnan et al., 2016) have been used. These networks have been trained and validated separately using the augmented dataset. At first, the transfer learning approach has been implemented to train the classification models. Initially, all the layers were frozen and the weights corresponding to training on the *ImageNet* dataset (Deng et al., 2010) were used to validate the networks on the augmented dataset. Subsequently, the Edisonian approach was followed by freezing and unfreezing different layers. For frozen layers, weights corresponding to training on the *ImageNet* dataset were used while the non-frozen layers were trained to the training set. Figures 11 and 12 show the architectures along with the frozen and unfrozen layers for *VGG16* and *InceptionV3* networks, respectively, that have given the maximum validation accuracy on the augmented dataset where grey boxes indicate the frozen layers. In the case of *VGG16*, the first four convolution layers have been frozen. This is followed by three repeating units of three convolution layers separated by a max pool layer. For each of these three units, a general pattern of freezing the middle convolution layer has been observed. The remaining two fully connected layers and softmax layers have been trained on the augmented dataset. In *InceptionV3*, the first five convolution layers have been frozen. The layers at the end in the form of fully connected and softmax have been trained to our dataset. In between, we have three different types of inception modules (L_1_, L_2_ and L_3_) where convolution layers have been frozen at random based on a trial-and-error approach. In the case of *Xception*, transfer learning did not prove to be efficient and all the layers were trained on the augmented dataset.

For each of these networks, the batch size is kept 2827 which is equal to the size of the training set. This ensures that at every iteration during training, all the entire training set used. The batch size is a hyperparameter that defines the number of samples to work through before updating the internal model parameters. 2,827 is the number of images present in the training set. Batch size has been kept equal to the size of the training set to ensure that the network encounters all the images before updating the parameters for the next epoch. This ensures a stable error gradient and a stable convergence for the learning algorithm. Four different optimizers were used for each of the three networks, *Nesterov Accelerated Gradient (NAG)* (Dozat, 2016), *Adagrad* (Lydia and Francis, 2019), *RMSprop* (Mukkamala and Hein, 2017), and *ADAM* (Zhang and Sabuncu, 2018). Training has been performed separately considering these four optimizers for all three networks. Hyperparameter tuning was performed for each of these optimizers for all three networks to determine the optimum value of learning rate. Loss function is set as *categorical cross entropy (CCE)* (Zhang and Sabuncu, 2018). The best validation accuracies by validating on the test set obtained for *VGG16*, *InceptionV3* and *Xception* considering the discussed architectures are 0.8313, 0.8688 and 0.8594 respectively.

#### Feature engineering approach

Since*InceptionV3* has given the maximum validation accuracy among the three pre-trained networks, it is used as a feature extractor on the training and test sets separately. This gives 12,289 features per image. This feature set corresponding to the training set is used to train two machine learning classifiers, random forest (RF) (Jin et al., 2020) and MLP (Gardner and Dorling, 1998). The F1 scores of classifications as obtained on the feature set corresponding to the test set for RF and MLP are 0.488 and 0.598 respectively. To improve upon the classification results, a meta-heuristic optimization algorithm, BRDA (Fathollahi-Fard et al., 2020), has been deployed on the feature set of the training set. The mating process ensures a competitive evolution at each stage of the algorithm where existing features are combined to produce new generations which are distinct from the parents with better fitness for a given optimization. This combination is important to extract highly non-linear interactions between features. BRDA reduces the size of the feature vector to 6072 features per image. The indices of the features retained in the optimized feature set corresponding to the training set is used to optimized the feature set of the test set. Following this feature selection, RF and MLP have been trained on the optimized feature set of the test set and the F1 scores on the optimized test feature set for RF and MLP are 0.963 and 0.969 respectively.

#### Binary red deer algorithm

Red Deer Algorithm (RDA) is a recently proposed nature-inspired meta-heuristic optimization algorithm (Fazliet al., 2019) which is derived from the mating behaviour of a sub-species of red deer known as Scottish red deer. During a breeding season, the male red deer (RD) begin the mating ritual by roaring which attracts the female counterparts called hinds. These male RDs' are categorized as commanders, ones having higher roaring intensity, and the rest are called stags. Every commander forms a harem, a group of hinds that mate with that particular commander. Harem size depends on the power of the commander defined by its fitness value. Besides, a commander also can mate with hinds belonging to other harems. Stags, on the other hand, mate randomly with their nearest hinds. This phenomenon of mating ensures a competitive evolution at each stage of the algorithm which explores the entire space of RDs'. In this work, we aim to use a binarized form of RDA called Binary Red Deer Algorithm (BRDA) for feature selection to choose an optimized subset of features from the whole set of features obtained from the augmented dataset using InceptionV3. The aim here is to maximize the classification accuracy simultaneously minimizing the number of features. Therefore, in this work, feature selection is modelled as a binary optimization problem, where the solutions are limited to {0,1}. In the BRDA, we first randomly initialize a vector of real numbers called RD of size m, the total number of features in the feature set.

(Equation 1) $$RD=\left[X_{1},X_{2},X_{3},\ldots ,X_{m}\right]$$

RD is converted into a binary vector (BRD) comprising only 0 and 1 using the Sigmoid function shown in Equation (2). Here 1 indicates that the corresponding feature is selected in the feature subset and vice versa for 0. The real values of RD are converted into binary values using a threshold of 0.5 as expressed in Equation (3).

(Equation 2) $$S\left(x\right)=\frac{1}{1+e^{-x}}$$

(Equation 3) $$X_{i}=\left\{ \begin{array}{ll} 1 & if\;S\left(X_{i}\right)>0.5\\ 0 & if\;S\left(X_{i}\right)\leq 0.5 \end{array}\right.$$

where *i*∈[1,*m*]. The quality of the RDs at every iteration of the algorithm is evaluated by a fitness function as expressed in Equation (12).

#### Initialization of RD population

At first, an RD population of size N is initialized randomly. Based on fitness values, the top RDs represent the males N_1_, and the rest of the RDs represent the hinds N_2_. The fraction of the RD to be considered as male is a hyperparameter for BRDA and needs to be specified manually.

#### Roaring male RDs

To successfully roar and attract hinds, the male RDs may change their positions according to Equation (4). If the fitness value of the male RDs at the new position is better than that in the original position, then the position of the RD is updated and roaring is considered to have been successful. Otherwise, theold position is retained.

(Equation 4) $$new=\left\{ \begin{array}{l} old+a_{1}\times \left(\left(upper-lower\right)\times a_{2}+lower\right)\;if\;a_{3}\geq 0.5\\ old-a_{1}\times \left(\left(upper-lower\right)\times a_{2}+lower\right)\;if\;a_{3}<0.5 \end{array}\right.$$

Here,old is the original position of the male RD whereas new is the position to which a male RD moves during the roaring procedure. a_1_, a_2_ and a_3_ are randomly generated numbers from a uniform distribution of 0 and 1 while upper = 1 and lower = -1 are the upper and lower bounds of the search space of the entire RD population.

#### Distinguishing commanders from stags

Among the male RDs, the top N_3_ are selected as commanders according to Equation (5)

(Equation 5) $$N_{3}=floor\left(\gamma \times N_{1}\right)$$

where *γ*∈[0,1] is a hyperparameterwhich is to be specified manually. Each of these commanders competes with the N_1_-N_3_number of stags randomly and two new solutions, New_1_ and New_2_ are generated as expressed in Equations (6) and (7) respectively. The position of the commander is updated using the solution which results in the best fitness value among the commander, the stag, and the two new solutions.

(Equation 6) $$New_{1}=\frac{Commander+Stag}{2}+b_{1}\times \left(\left(upper-lower\right)\times b_{2}\right)+lower)$$

(Equation 7) $$New_{2}=\frac{Commander+Stag}{2}-b_{1}\times \left(\left(upper-lower\right)\times b_{2}\right)+lower)$$

Commander + Stag represents addition of the two vectors corresponding to commander and stag respectively. b_1_ and b_2_ are generated using a uniform distribution function in [0,1].

#### Formation of harems

Since this is a minimization problem, better quality of solution is determined by a lower fitness value. Therefore, we find the power of each commander according to Equation (8)

(Equation 8) $$P_{j}=F-f_{j}$$

where P_j_ and f_j_ are the power and fitness value of the *j*th commander respectively and *j*∈[1,*N*_3_]. F is the sum of fitness values of all commanders. Equation (9) represents the fraction of the total number of hinds that form a harem with a particular commander.

(Equation 9) $$N_{4_{j}}=floor\left(P_{j}\times N_{2}\right)$$

where $N_{4_{j}}$ is the number of hinds that belongs to the *j*th harem.

#### Mating of commanders

In each harem, all hinds mate with the respective commander to produce offspring according to Equation (10).

(Equation 10) $$offspring=\frac{Commander+Hind}{2}+\left(upper-lpwer\right)\times c$$

Here c is a randomly generated number between 0 and 1. Besides, all the commanders can mate with hinds from all other harems. Consequently, a new population of RDs is generated which are stored in *offspring pool*.

#### Mating of stags

Each stag mates with its nearest hind irrespective which of harem the hind belongs to. The distance between a stag and all hinds is calculated by Equation (11).

(Equation 11) $$d_{k}=\sqrt{\sum_{k\in N_{2}}{\left(stag_{k}-hind_{k}^{j}\right)}^{2}}$$

where d_k_ is the distance between a stag and the *j*th hind. The hind at the minimum distance is selected for mating, which takes place according to Equation (10), with the stag replacing the male commander. The offspring formed in this process are added to the *offspring pool*.

#### Selection of next generation

After the mating process is completed, the offspring from the *offspring pool* are shuffled with the original population. The top N RDs are selected according to fitness values as the next generation and the rest of the solutions are discarded.

#### Terminating the BRDA

The process is stopped when *iter*number of iterations are completed. The RD with the best fitness value in the final generation represents the optimized feature subset which is used to train the RF and MLP classifiers.

#### Fitness evaluation

Fitness quantifies the quality of the BRDA solution. A learning algorithm based on an RF classifier is used to evaluate the performance of a particular feature subset along with the whole feature set. The fitness function consists of two components: classification accuracy and the number of features. Our objective is to achieve the highest classification accuracy minimizing the number of features. Higher classification accuracy and fewer features imply a low fitness value. The fitness function is shown in Equation (12)

(Equation 12) $$Fitness=\kappa \times \frac{\left|Selected\right|}{\left|Total\right|}+\left(1-\kappa \right)\times \varphi$$

where |Selected| is the number of features in the selected feature subset, |Total| is the total number of features of the dataset, *φ* is the classification error of the feature subset, and *κ*∈[0,1] indicates the relative weight assigned to the number of features and the classification error.

The time complexity of BRDA is expressed as O(iter∗N^2^∗(t+m)) where t is the time complexity in calculating the fitness of a particular RD using the RF classifier.

**Figure:**
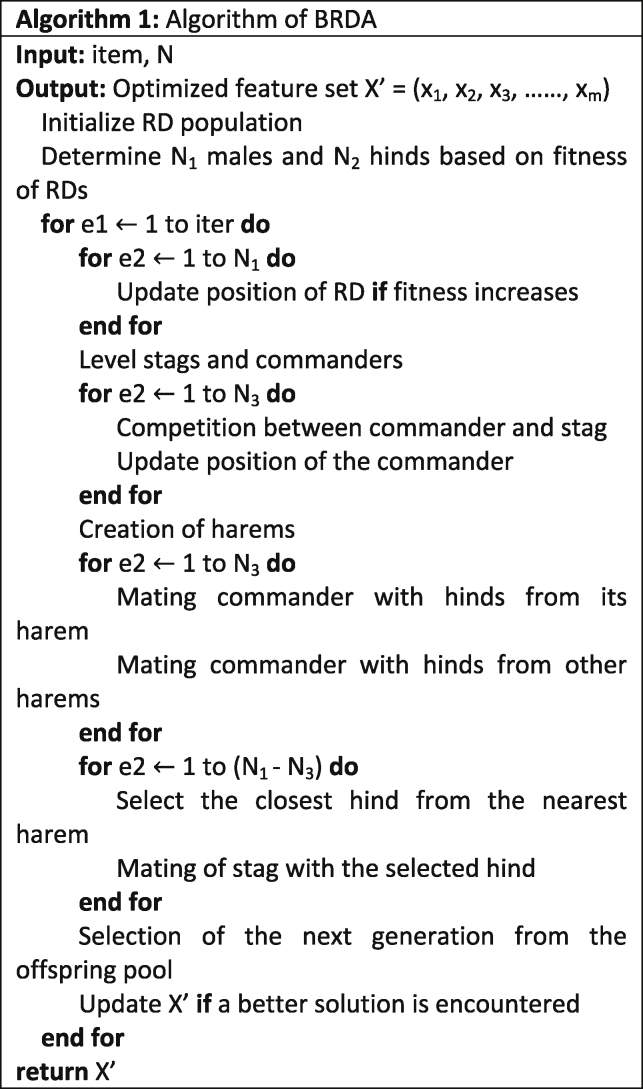

## Data Availability

Data: The dataset has been built using the Dissemination of Information Technology for Promotion of Materials Science (DoITPoMS), a web-based initiative that started in the Materials Science and Metallurgy Department at Cambridge University (Barber et al., 2007) DoITPoMS comprises a collection of micrographs covering a wide range of specimen types like ceramic, metal or alloy, device, composite, polymer, foam, etc., and microscopy techniques like optical micrography, SEM, or TEM. Information related to chemical composition and processing technique is also available as metadata for every microstructure. Additionally, in some cases, microstructures corresponding to a particular specimen are available at different magnifications. We have collected a total of 577 micrographs across 21 different alloy systems and based on their morphologies, we have divided them into two categories, dendritic and non-dendritic comprising 133 and 444 images respectively. Using these 577 micrographs, we have prepared training and test sets. The training set consists of 93 dendritic and 310 non-dendritic micrographs while the test set comprises 40 dendritic and 134 non-dendritic micrographs. This proposed work aims to develop a binary image classification model that can distinguish microstructures having dendritic morphology from non-dendritic ones. Figure 10 shows some example images used to build the proposed binary classification models. The dataset is available at https://github.com/stochasticmaterialism/Dendritic-Non-Dendritic-Classification. Code: The code developed for this paper is available at https://github.com/stochasticmat}\\\textbf{erialism/Dendritic-Non-Dendritic-Classification.
